# Systemic inflammation mediates environmental polycyclic aromatic hydrocarbons to increase chronic obstructive pulmonary disease risk in United States adults: a cross-sectional NHANES study

**DOI:** 10.3389/fpubh.2023.1248812

**Published:** 2023-11-21

**Authors:** Yingqi Xiao, Li Zhang, Hu Liu, Wei Huang

**Affiliations:** ^1^Department of Pulmonary and Critical Care Medicine, Dongguan Tungwah Hospital, Dongguan, China; ^2^Department of Orthopedics, The Sixth Affiliated Hospital, School of Medicine, South China University of Technology, Foshan, China

**Keywords:** polycyclic aromatic hydrocarbons, chronic obstructive pulmonary disease, systemic immune-inflammation index, mediation analyses, NHANES

## Abstract

**Introduction:**

This study explored the relationship between environmental polycyclic aromatic hydrocarbons (PAHs) and Chronic obstructive pulmonary disease (COPD), and identified systemic inflammation as a mediator of the increased risk of COPD from PAHs.

**Methods:**

Data were obtained from 60,936 middle-aged and older Americans recruited in the National Health and Nutrition Examination Survey 2005–2016. Environmental PAHs were measured in terms of urinary concentrations of PAHs metabolites (NAP: 1-hydroxynaphthalene, FLU: 2-hydroxyfluorene, PA: 1-hydroxyphenanthrene, and PYR: 1-hydroxypyrene). We used multifactor logical analysis to figure out the link between PAHs and COPD, and the non-linear relationship was examined using Restricted cubic spline. Spearman correlation analysis was utilized to analyze the connection between PAHs and systemic immune-inflammation index (SII).

**Results:**

The results showed that the COPD population had higher NAP (3.550 vs. 3.282, *p* < 0.001), FLU (2.501 vs. 2.307, *p* < 0.001), PA (2.155 vs. 2.082, *p* = 0.005), and PYR (2.013 vs. 1.959, *p* = 0.008) levels than non-COPD population. In unadjusted logistics analysis, the risk of COPD with log NAP was higher [OR = 1.461, 95% CI (1.258–1.698), *p* < 0.001]. Upon taking into account, confounders like sex, age, race, and log NAP still increased a possible COPD risk [OR = 1.429, 95% CI (1.224–1.669), *p* < 0.001]. Similarly, FLU, PA, and PYR significantly increased the risk of COPD (all OR > 1, *p* < 0.05), both unadjusted and adjusted. Furthermore, Restricted cubic spline demonstrated a strong link between PAHs levels and COPD risk (*p* < 0.05). Additionally, a Spearman correlation analysis revealed a favorable association between log FLU and log SII (*R* = 0.43, *p* = 0.006), while NAP, PA, and PYR levels were not associated with log SII (all *p* > 0.05). Ultimately, the mediating effect analysis revealed a mediating effect capacity of 5.34% for the SII-mediated association between FLU and COPD.

**Conclusion:**

The findings suggest that the risk of COPD is significantly increased when environmental PAHs exposure is at high levels, and that systemic inflammation may be involved in the process.

## 1 Introduction

Chronic obstructive pulmonary disease (COPD), a prevalent chronic airway disease, is distinguished by persistent respiratory symptoms and irreversible airflow limitation (1). As a heterogeneous disease, COPD develops frequently in adults over 40 and is intimately linked to environmental pollution, especially with exposure to toxic gases (2, 3). Currently, as environmental pollution aggravates and the population ages, a growing number of people are suffering from COPD. Meanwhile, high morbidity and mortality rates are associated with COPD, which imposes a significant disease burden on the international community (4).

Polycyclic aromatic hydrocarbons (PAHs) are a collection of compounds that are produced through the incomplete combustion of oil, coal and natural waste, gas, and other organic materials. PAHs have been linked to a variety of health risks (5, 6). PAHs can be derived from a broad variety of sources, including but not limited to vehicle exhaust, asphalt, coal tar, wildfires, agricultural burning, food that has been grilled, and tobacco smoke (7, 8). After entering the human body, PAHs are metabolized by means of the peroxidase pathways, and produce carcinogens such as reactive diol epoxides, o-quinones, and free radical cations (9). Although PAHs are known to be a health hazard, the association between PAHs and COPD has not been fully elucidated so far, and the specific pathways by which PAHs affect COPD are not yet known.

A recently developed inflammatory indicator is known as the systemic immune-inflammation index (SII) that simply combines the functions of neutrophil, platelet, and lymphocyte counts; due to its high stability, it can effectively reflect systemic inflammation (10, 11). SII is strongly associated with prognosis and survival of cancer (11), endocrine disease (12), and kidney disease (10). SII and COPD are closely related, and previous studies have confirmed that blood SII is associated with COPD exacerbations (13). Higher SII levels in COPD patients also significantly increase the risk of death (14). Besides, SII mediates the development of many related disorders. For instance, You et al. (15) found that SII mediated the association between sedentary behavior and sleep disorders. Yin et al. (16) also demonstrated that elevated SII levels slightly mediated the association between sleep disturbance and depression. Furthermore, additionally, SII might act as a mediator between fetal famine exposure on the development of cardiovascular disease in adulthood (17).

Apparently, there is a paucity of direct robust evidence supporting the link between PAHs and the risk of COPD, and there is little known about whether PAHs progress by increasing systemic inflammation and thereby influence the development of COPD. Therefore, we desire to investigate the link between PAHs and COPD in middle-aged and older adults among participants recruited by the National Health and Nutrition Examination Survey (NHANES). In addition, we included SII as a mediator to assess whether SII was involved in the connection between COPD and PAHs in adults over 40 years old.

## 2 Materials and methods

### 2.1 Source of subjects

The NHANES is a survey that is carried out by the National Center for Health Statistics in conjunction with the Centers for Disease Control and Prevention. Since it stores data from the civilian population of the United States, it is nationally representative. The Ethics Review Committee gave their stamp of approval to the protocol for collecting the data, and before they were questioned or evaluated, each participant in the study gave their informed consent. Hence, the study did not require additional informed consent as well as ethical endorsement. The NHANES public data file from 2005 to 2016 was used to construct the dataset in this study, and this research was carried out with the assistance of a total of 60,936 people.

### 2.2 PAHs metabolites measurement

The main exposure variable in this study was PAH with reference to the former studies (8), we used urine PAHs to measure the level of PAHs in individuals. The NHANES database participants’ urine PAHs were first extracted in (ng/L), including 1-hydroxynaphthalene (NAP), 2-hydroxyfluorene (FLU), 1-hydroxyphenanthrene (PA), and 1-hydroxypyrene (PYR). The measurement of PAHs was accomplished principally through the enzymatic digestion of urine, followed by extraction, derivatization, and examination by means of capillary gas chromatography coupled with high-resolution mass spectrometry. In brief, we used isotopic dilutions of carbon-13-labeled internal standards. The ions of each analyte and each carbon-13 labeled by internal standard were monitored and the abundance of each ion was measured. In this way, the levels of PAHs were measured by the analysis of certain urine analytes. Since urine PAH concentrations are skewed distribution data, the log10 log transformation was taken (18). The specific measurement method is described in detail in the NHANES at https://www.cdc.gov/nchs/nhanes/index.htm.

### 2.3 COPD definition

Referring to previous studies (19), if the following conditions are met, it can be diagnosed as COPD: (1) FEV1/FVC < 0.7 after inhaling bronchodilators; (2) concerning questions from the MCQ questionnaire “mcq160g” or “mcq160p”: “ever told you had emphysema,” answering “yes” was considered COPD; and (3) Over 40 years of age, long-term use of selective phosphodiesterase-4 inhibitor, mast cell stabilizer, leukotriene modulator and inhaled corticosteroids, and a history of smoking or chronic bronchitis.

### 2.4 Mediating variable

The mediating variable in this study was systemic inflammation, which was assessed based on SII. SII = P × N/L (P: peripheral platelet, N: neutrophil, L: lymphocyte, in ×10^9^/L) (10). A higher SII represents a higher level of systemic inflammation in the study population. Since SII is also skewed distribution data, log_10_ log transformation was also taken.

### 2.5 Other covariates

Patient baseline information variables were selected primarily in terms of demographics, social factors, lifestyle habits, and comorbidities. As well as demographics such as gender, age, and race, social factors such as education level and marriage were also considered. Behavioral data were collected on smoking and alcohol consumption as part of the study. Also, variables associated with medical comorbidities, such as BMI, hypertension, diabetes, and hyperlipidemia, were collected. In this study, smoking was not considered as a covariate in the subsequent logistic regression because of the high correlation between smoking and the level of exposure to PAHs (20). Missing data were filled by interpolation.

### 2.6 Statistical analysis

Quantitative data were verified using the *t*-test or rank sum test, and the categorical variable data were verified using the χ^2^ test to examine variations in cohort characteristics between outcome variable groups. Determine the covariates that need to be adjusted, including age, race, sex, and BMI. Preliminary analysis included multifactorial logistic regression to investigate the relationship between PAHs and CPOD. Restricted cubic spline was used to examine the nonlinear relationship between the COPDs variables and PAHs. An analysis of Spearman correlations was performed to assess the relationship between PAHs and SII. Finally, In order to identify the potential mediating effects of mediating variables on the association between PAHs and COPD, we used a mediation effect model (21). R.3.5.2^1^ was used for all data analysis. A sample size calculation was not performed ex ante, just based on available data. *p* < 0.05 was set as the level of statistical significance.

## 3 Results

### 3.1 Characteristics of NHANES participants

**Figure 1:**
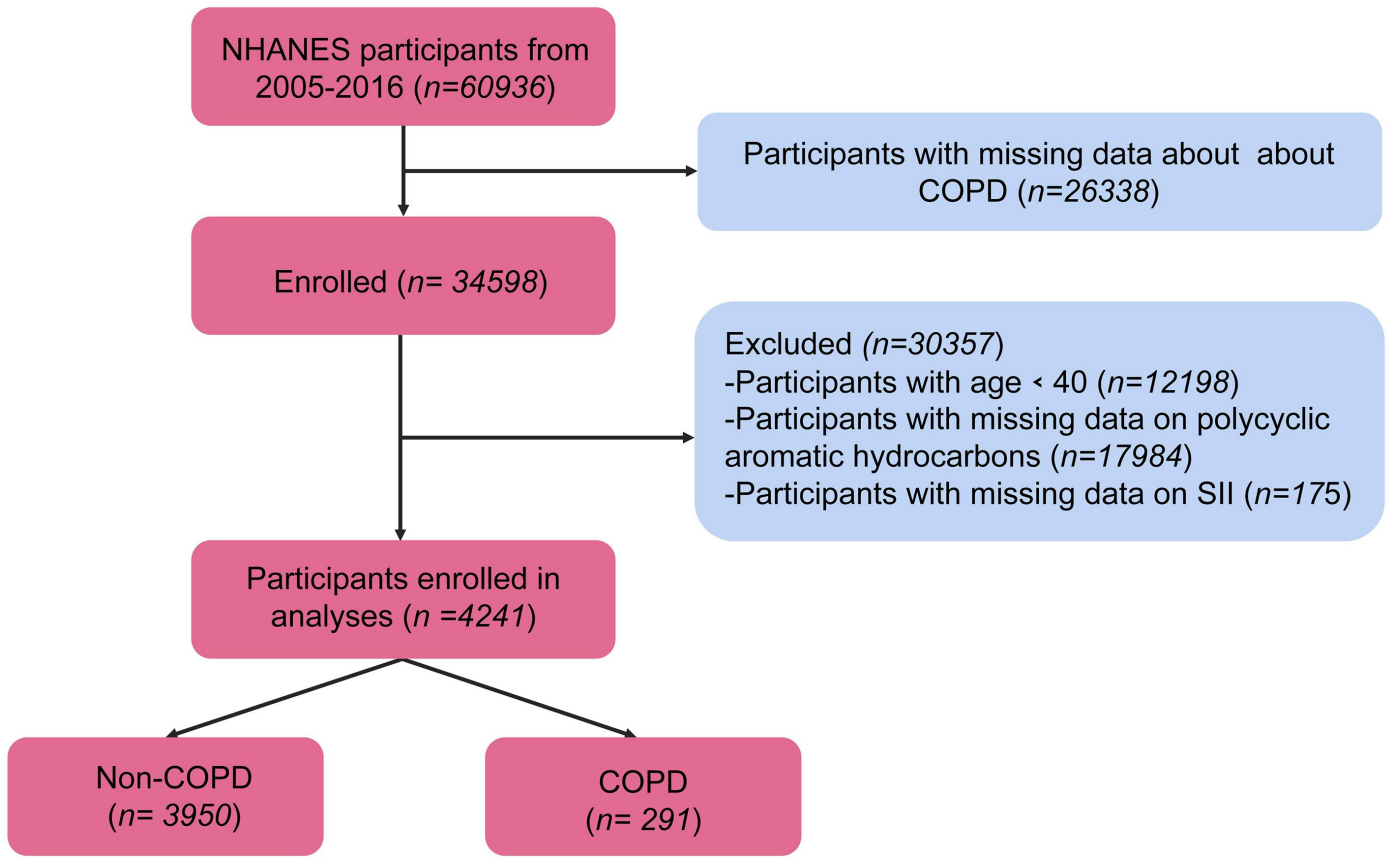
Flow diagram of the screening and selection process.

Initially, a total of 60,936 participants participated in the survey. Among them, 4,241 people were included in the final analysis. Ultimately, excluding data without outcomes or exposures, 4,241 middle-aged and older United States participants (COPD: 291, and non-COPD: 3950) were included (Figure 1).

Table 1 shows the basic characteristics for study participants. Participants with COPD numbered 291 and those non-COPD numbered 3,950 (Table 1). With increasing age, COPD prevalence increased, specifically in participants of 40–49 years (14.8%), 50–59 years (19.9%), and ≥ 60 years (65.3%) (Table 1). Also, there was a significant difference between the COPD and non-COPD groups in terms of the proportion of age ≥ 60 years [(65.3%) vs. (47.7%), *p* < 0.001] and the proportion of men [(57.7%) vs. (49.6%), *p* = 0.007] were significantly higher in the COPD group than in the non-COPD group (Table 1). Divorced, separated, and widowed [(35.7%) vs. (29.1%), *p* = 0.040] individuals were more common in the COPD group in comparison to non-COPD patients, while in terms of higher education [(70.4%) vs. (71.4%), *p* = 0.040], the two groups were not significantly different (Table 1). Furthermore, there was a significant increase in “Former” smoking [(47.8%) vs. (29.3%), *p* < 0.001] and “Now” smoking [(34.7%) vs. (18.1%), *p* < 0.001] behavior in the COPD population compared with the non-COPD population. Similarly, COPD patients consumed more alcohol than non-COPD patients [(88.3%) vs. (79.1%), *p* < 0.001; Table 1]. In terms of phlebotomy, there was no significant difference in Alanine aminotransferase (AST) and Aspartate aminotransferase (ALT) between the two groups (all *p* > 0.005, Table 1). In contrast, estimated glomerular filtration rate (eGFR) levels were lower in the COPD population compared to those without COPD population [(78.0) vs. (86.9), *p* < 0.001; Table 1]. Furthermore, for medical comorbidities, there was a greater prevalence of hypertension [(68.7%) vs. (54.6%), *p* < 0.001] and diabetes [(29.6%) vs. (23.8%), *p* = 0.026] among people with COPD than among people without COPD (Table 1). BMI and hyperlipidemia did not differ significantly between these two groups (all *p* > 0.005, Table 1).

**Table 1:** Characteristics of participants enrolled in study.

Characteristic	Non-COPD (*n = 3,950*)	COPD (*n = 291*)	*p* value
Age (years)			<0.001
40–49	1,085 (27.5%)	43 (14.8%)	
50–59	981 (24.8%)	58 (19.9%)	
≥60	1,884 (47.7%)	190 (65.3%)	
Male sex	1,958 (49.6%)	168 (57.7%)	0.007
Race			<0.001
Hispanic	977 (24.7%)	32 (11.0%)	
Non-Hispanic white	1,967 (49.8%)	204 (70.1%)	
Non-Hispanic black	765 (19.4%)	43 (14.8%)	
Other	241 (6.1%)	12 (4.1%)	
Education beyond high school	2,819 (71.4%)	205 (70.4%)	0.738
Marital status			0.040
Never married	332 (8.405%)	18 (6.2%)	
Married or living with partner	2,467 (62.5%)	169 (58.1%)	
Divorced, separated, or widowed	1,151 (29.1%)	104 (35.7%)	
BMI (kg/m^2^)			0.176
<25	1,003 (25.4%)	88 (30.2)%	
25–29	1,382 (35.0%)	98 (33.7%)	
≥30	1,565 (39.6%)	105 (36.1%)	
ALT (U/L)	22.0 (17.0–28.0)	21.0 (16.0,28.0)	0.257
AST (U/L)	24.0 (20.0–28.0)	23.0 (21.0,29.0)	0.67
eGFR (mL/min per 1.73 m^2^)	86.9 (71.6–99.7)	78.0 (63.8,94.5)	<0.001
Alcohol user	3,126 (79.1%)	257 (88.3%)	<0.001
Smoker			<0.001
Never	2,079 (52.6%)	51 (17.5%)	
Former	1,157 (29.3%)	139 (47.8%)	
Now	714 (18.1%)	101 (34.7%)	
Diabetes	939 (23.8%)	86 (29.6%)	0.026
Hypertension	2,156 (54.6%)	200 (68.7%)	<0.001
Hyperlipidemia	3,097 (78.4%)	242 (83.2%)	0.056
PAHs			
Log NAP	3.282 (2.898–3.834)	3.550 (3.087–4.051)	<0.001
Log FLU	2.307 (2.009–2.702)	2.501 (2.138–2.961)	<0.001
Log PA	2.082 (1.806–2.354)	2.155 (1.880–2.433)	0.005
Log PYR	1.959 (1.695–2.267)	2.013 (1.747–2.362)	0.008

Categorical data are displayed as *n* %. Non-normal distribution data are displayed as median (Q1–Q3). χ^2^ analysis is used to test significance between groups for categorical data. Kruskal Wallis rank sum test is used to test significance between groups for non-normal distribution data. BMI, Body mass index; eGFR, Estimated glomerular filtration rate; ALT, Alanine aminotransferase; AST, Aspartate aminotransferase; NAP, 1-hydroxynaphthalene; FLU, 2-hydroxyfluoren; PA, 1-hydroxyphenanthrene; and PYR, 1-hydroxypyrene.

### 3.2 Association between PAHs levels and COPD risk

According to Table 1, COPD patients had higher NAP [(3.550) vs. (3.282), *p* < 0.001], FLU [(2.501) vs. (2.307), *p* < 0.001], PA [(2.155) vs. (2.082), *p* = 0.005], and PYR [(2.013) vs. (1.959), *p* = 0.008] levels compared with non-COPD patients.

Also, in unadjusted logistics analysis (Table 2), the risk of developing COPD was higher with the growth of log NAP. Each 1 unit increase in log NAP meant a 1.461-fold increase in COPD risk [OR = 1.461, 95% CI (1.258–1.698), *p* < 0.001]. Taking into account sex, age, race, and BMI as covariates, with the increase of log NAP, the risk of developing COPD remained high [OR = 1.429, 95% CI (1.224–1.669), *p* < 0.001; Table 2]. Similarly, an increased risk of COPD was associated with FLU, PA, and PYR (all OR > 1, *p* < 0.05; Table 2), both unadjusted and adjusted.

**Table 2:** Odds ratios for associations between PAHs and COPD.

OR (95% CI)	Un-adjusted	*p* value	Adjusted	*p* value
Log NAP	1.461 (1.258–1.698)	<0.001	1.429 (1.224–1.669)	<0.001
Log FLU	1.804 (1.461–2.227)	<0.001	2.072 (1.654–2.597)	<0.001
Log PA	1.403 (1.055–1.864)	0.020	1.417 (1.056–1.901)	0.027
Log PYR	1.357 (1.054–1.747)	0.018	1.677 (1.290–2.180)	<0.001

Adjusted for age, race, sex, and BMI.

**Figure 2:**
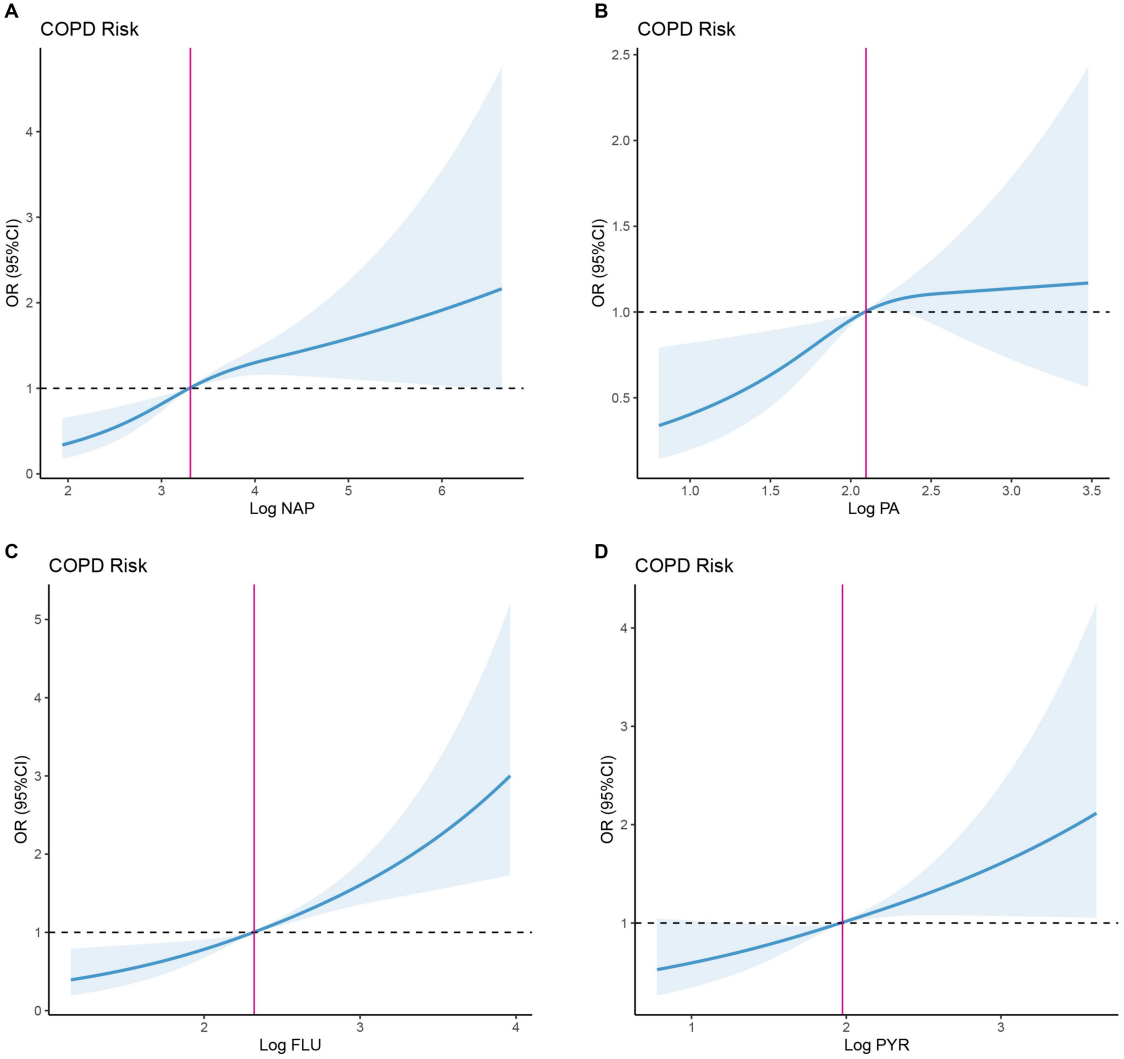
Dose–response association between PAHs and OR of COPD. **(A)** Dose–response association between NAP and OR of COPD. **(B)** Dose–response association between FLU and OR of COPD. **(C)** Dose–response association between PA and OR of COPD. **(D)** Dose–response association between PYR and OR of COPD. NAP, 1-hydroxynaphthalene; FLU, 2-hydroxyfluorene; PA, 1-hydroxyphenanthrene; and PYR, 1-hydroxypyrene.

Moreover, we analyzed the non-linear relationship between PAHs and COPD prevalence risk based on RCS. Overall, risks of COPD were positively correlated with PAH levels (Figure 2). Specifically, COPD risk tended to increase with the rise of log NAP, with the inflection point (OR = 1) occurring at log NAP = 3.309 (Figure 2A; Table 3), and this trend was not significant in the period before and after the inflection point (*p* > 0.05). Similarly, there was a similar intra-segmental variation around the inflection point for log PA (Figure 2B; Table 3). However, there was no significant trend in COPD risk with the increase of log FLU in the pre-inflection point segment, but a significant trend in COPD risk with the increase of log FLU in the post-inflection point segment (*p* < 0.05; Figure 2C; Table 3). Log PYR, conversely, was significantly correlated with COPD risk in both pre-and post-inflection point segments (*p* < 0.05; Figure 2D; Table 3).

**Table 3:** Threshold effect analysis of PAHs on odds ratios for COPD using the restricted cubic spline model.

ITEM	Inflection point (OR = 1)	Adjusted β (95%CI)	*p* value
Log NAP	3.309		
< Inflection point		1.745 (0.866–3.518)	0.119
≥Inflection point		1.211 (0.942–1.555)	0.135
Log FLU	2.321		
< Inflection point		1.555 (0.681–3.550)	0.295
≥Inflection point		1.915 (1.303–2.816)	<0.001
Log PA	2.095		
< Inflection point		1.660 (0.749–3.678)	0.212
≥Inflection point		1.314 (0.623–2.063)	0.681
Log PYR	1.976		
< Inflection point		2.226 (1.064–4.662)	0.034
≥Inflection point		1.722 (1.062–2.792)	0.027

β represented increased value of odds ratios for COPD when log PAHs increased by 1 unit. All was adjusted for age, race, sex, and BMI.

### 3.3 Association between PAHs levels and systemic inflammation

**Figure 3:**
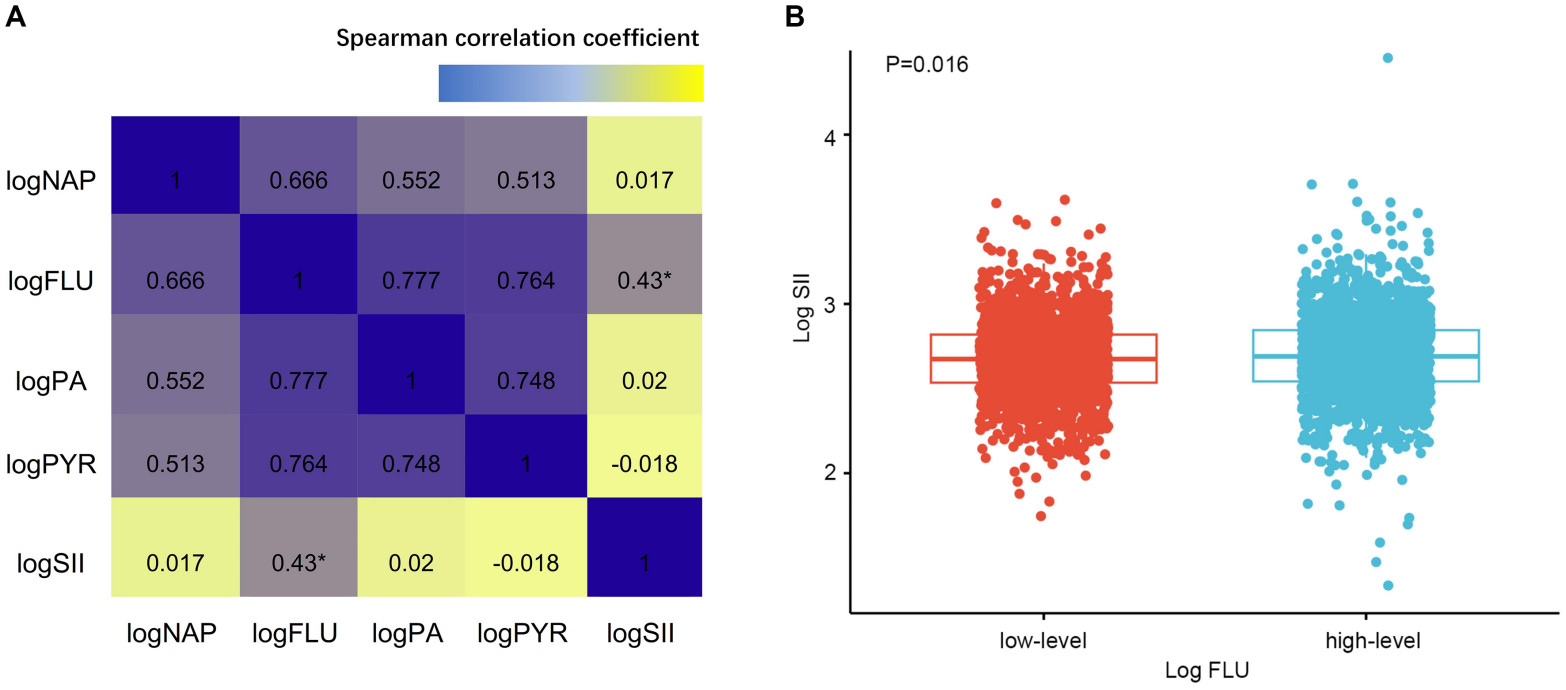
**(A)** Associations between PAHs and SII by Spearman correlation analysis. **(B)** Different SII levels of FLU. SII, Systemic immune-inflammation index. ^*^p < 0.005.

Besides, we explored the association between PAHs levels and SII. Spearman analysis revealed a positive correlation between log FLU and log SII in the overall study population (*R* = 0.43, *p* = 0.006, Supplementary Table S1; Figure 3A), while other types of PAHs exposure levels were not significantly correlated with log SII (all *p* > 0.05, Figure 3A). Furthermore, based on the log FLU median, participants were divided into two groups: high-log FLU and low-log FLU. As compared with the low-log FLU group, the high-log FLU group had significantly higher SII (*p* = 0.016; Figure 3B), suggesting that as log FLU levels increased, systemic inflammation levels also tended to increase.

### 3.4 Mediating effect analysis

**Figure 4:**
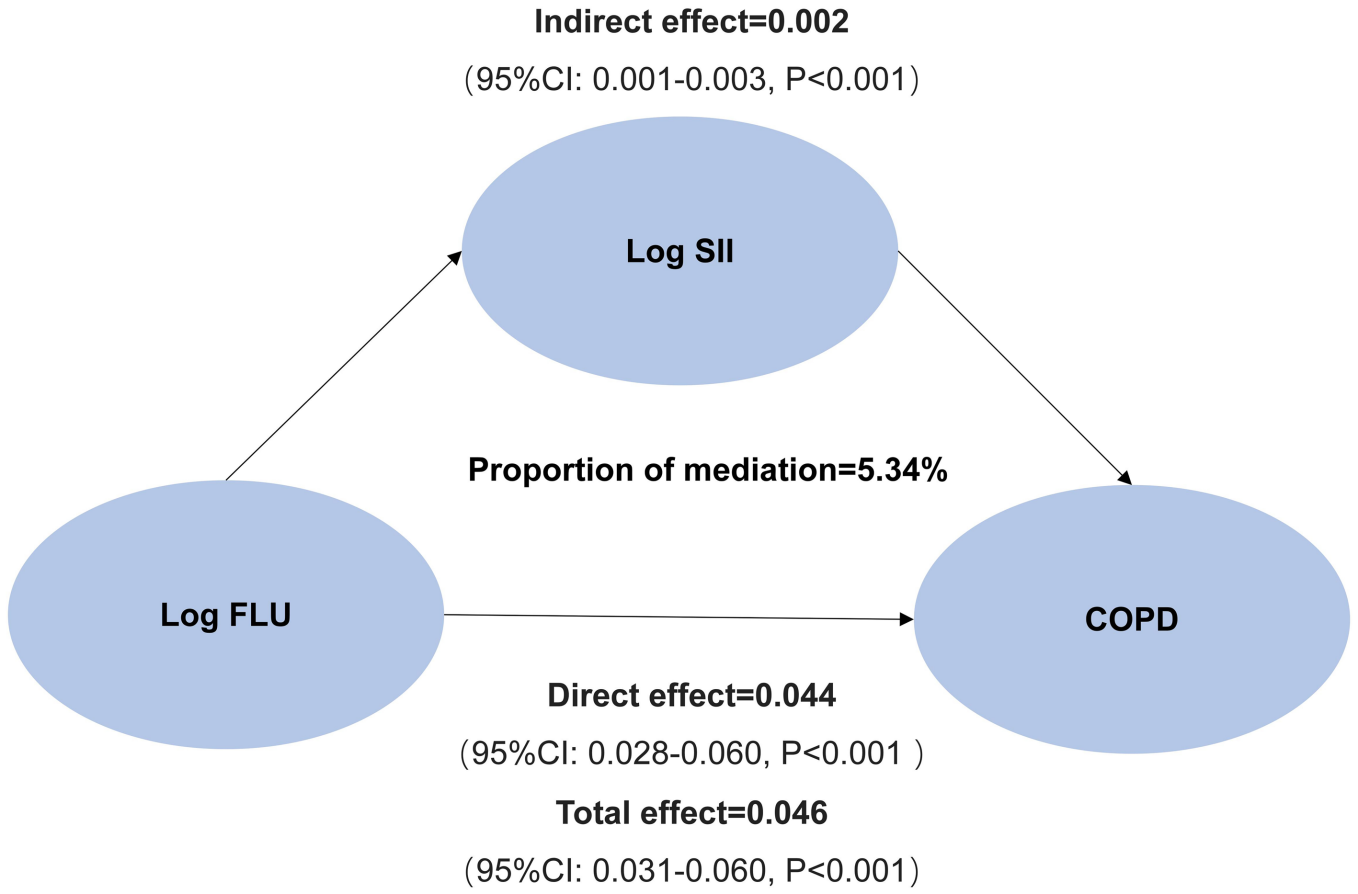
Mediation analysis of SII on the interaction between FLU and prevalence of COPD.

Finally, we analyzed the mediating effects of log FLU, log SII, and COPD risk after covariate adjustment. As shown in Figure 4, an increase in log FLU increased the risk of COPD, with a total effect value was 0.046 (95% CI: 0.031–0.060, *p* < 0.001), including a direct effect value was 0.044 (95% CI: 0.028–0.060, *p* < 0.001). In addition, the indirect effect value for log FLU leading to COPD prevalence risk via log SII was 0.002 [(95% CI (0.001–0.003), *p* < 0.001); Figure 4].

To conclude, the mediating effect of systemic inflammation mediating the association between FLU and COPD prevalence risk produced a mediating effect capacity of 5.34% (Figure 4).

## 4 Discussion

In general, PAHs are mainly exposed to people through breathing indoor and outdoor polluted air and smoking (22). PAHs exposure is not a single effect, but often has multiple pathways, depending on the level of exposure (duration of time), the concentration of exposure to PAHs, toxicity, and routes of exposure, such as inhalation, ingestion, or dermal contact, as well as factors influencing human age, habits of living, and health status (23).

During large cross-sectional study of middle-aged and older adult people in the United States, higher concentrations of environmental PAHs were positively correlated with the risk of COPD. Previous studies have also shown similar results. For instance, Peng et al. (18) found that PAHs, both present and continuous exposure, contributed significantly to COPD risk, particularly NAP (OR: 1.83, *p* < 0.005) and FLU (OR: 2.29, *p* < 0.005). Furthermore, we verified that COPD patients are generally older male, and have a higher prevalence of smoking as well as alcohol consumption in accordance with previous research (18). Previous studies (18) have found that there was an independent association between lower BMI and COPD risk in nonsmokers. In contrast, the BMI of the two groups did not differ significantly, which might be attributed to errors caused by the period of data collection (NHANES 2007–2016), sample size (COPD: 500) of Peng et al. (18) not being exactly the same as ours, as well as our inclusion of a population restricted to >40 years. The main cause of chronic obstructive pulmonary disease is chronic airway inflammation and oxidative stress (24). PAHs can contribute to the activation of airway inflammation through Wnt5a-YAP/TAZ signaling, leading to acute lung dysfunction (25). Our study also found that FLU was positively associated with SII, and the mediating effect showed that SII mediated the association between FLU and COPD prevalence risk, but other types of PAHs exposure levels were not significantly associated with SII. As a result of its association with chronic bronchitis, FLU has been linked to various lung diseases (26), emphysema, asthma (27), lung infection, and COPD, which in line with our study’s findings (28).

Currently, research on the human hazards caused by PAHs primarily focuses on deformities, carcinogenicity, and neurotoxicity, while studies on the chronic lung inflammation caused by direct exposure to PAHs are relatively limited (29). Through mediating effects analysis, we found that FLU may significantly increase the risk of COPD in the population by increasing systemic inflammation. It has been shown that PAHs can increase oxidative stress and thus increase the risk of asthma in children (30). Therefore, it can be inferred that the oxidative stress caused by PAHs, represented by FLU, causes an elevated level of systemic inflammation and increases the risk of slow COPD disease in exposed populations.

Polycyclic aromatic hydrocarbons are primarily generated during the incomplete combustion of organic materials (31). PAHs are also present in certain manufactured goods such as dyes, plastics, and pesticides. Due to their widespread presence, human exposure to PAHs is almost inevitable, with inhalation and ingestion being the primary routes of exposure (31).

The impact of PAHs on human health is significant, particularly in relation to lung health. Inhalation of PAHs can lead to both acute and chronic respiratory conditions (32). Preventing or minimizing exposure to PAHs is crucial for lung health. This can be achieved through a multi-pronged approach. At an individual level, lifestyle changes such as quitting smoking, reducing consumption of charred foods, and avoiding exposure to vehicle exhausts can significantly reduce PAH exposure (33). From an occupational perspective, workers in high-risk industries should be provided with appropriate personal protective equipment and safety training (33). At a broader societal level, stricter regulation and enforcement of industrial emissions, promotion of cleaner sources of energy, and regular monitoring of air quality can help control the levels of PAHs in the environment (34).

2-hydroxyfluorene, as a type of PAH, has been associated with various health effects largely related to its ability to induce oxidative stress and inflammation. In our study, after adjusting for covariates such as age, sex, race, and BMI, log FLU had a positive effect on the risk of COPD prevalence, and the indirect effect of log FLU leading to the risk of COPD prevalence through log SII accounted for 5.34% of the risk of COPD prevalence, suggesting that SII is an important pathway for FLU to increase the risk of COPD prevalence, and that the overall effect of 5.34% of the risk of COPD prevalence due to FLU may be realized through the systemic inflammatory response realized. Although this mediating effect may appear relatively small, it is statistically significant and represents an important contribution to the overall effect of the disease in a complex disease pathology. According to Ferguson et al. (35) urinary PAH metabolites, which are biomarkers of internal PAH exposure, are associated with biomarkers of inflammation, angiogenesis, and oxidative stress in pregnant women. Although their study focuses on pregnant women, it demonstrates the potential of PAHs, including FLU, to induce systemic inflammation. Moreover, Cheng et al. (36) found that IL-22, a cytokine involved in immune response and inflammation, might be a potential mediator of associations between urinary PAH metabolites and health outcomes including fasting plasma glucose and type 2 diabetes. This study provides further evidence of the mechanism through which PAH exposure, including FLU, can lead to systemic inflammation. In a similar vein, Zhang et al. (37) showed that exposure to PAHs in outdoor air was associated with respiratory health, inflammation, and oxidative stress biomarkers in healthy young adults. Indeed, it remains unclear as to how FLU specifically affects systemic inflammation, but this finding also re-enforces the link between PAH exposure and inflammation and extends these findings to respiratory health, which is relevant to our research on COPD.

In light of these findings, we hypothesize that exposure to FLU may lead to systemic inflammation, as measured by SII, through the induction of oxidative stress and the production of pro-inflammatory cytokines. This increased systemic inflammation then may contribute to the development or exacerbation of COPD. However, the specific mechanisms through which FLU influences SII and COPD risk warrant further study.

Nonetheless, there are still some limitations to our study. At first, the present study is a cross-sectional study and cannot analyze the association between PAHs and COPD over time, which needs to be complemented by a longitudinal study. Besides, PAHs or SII can only be used as a qualitative diagnostic tool for COPD and cannot assess the extent of COPD as well as lung function. Therefore, the correlation between the dose of PAHs exposure and the severity of COPD needs to be elucidated by further experiments. Also, the present study is an association study and cannot explain causes and effects of COPD caused by PAHs. Furthermore, how PAHs affect COPD is very complex, and more basic validation of the relationship is needed, and future studies need to fully elucidate the specific molecular biological mechanisms of NAP and FLU-induced COPD.

## 5 Conclusion

In the study, we found a strong association between exposure to PAHs and COPD risk among a representative sample across the middle-aged and older adult people in United States and that there is a mediation effect, a process mediated through systemic inflammation. In particular, FLU has the potential to elevate SII and thereby increase the risk of COPD.

## Data availability statement

The original contributions presented in the study are included in the article/Supplementary material, further inquiries can be directed to the corresponding authors.

## Ethics statement

The studies involving humans were approved by Centers for Disease Control and Prevention in United States. The studies were conducted in accordance with the local legislation and institutional requirements. Written informed consent for participation was not required from the participants or the participants’ legal guardians/next of kin in accordance with the national legislation and institutional requirements.

## Author contributions

YX carried out the acquisition and interpretation of data and was the major contributor to drafting the manuscript. YX and LZ carried out the clinical partial data collection and analysis. WH and YX participated in drawing tables and diagrams. HL was responsible for correcting the language and grammar. WH was responsible for reviewing and revising some drawings and tables. HL and WH contributed to the ideas of the article and reviewed the manuscript. All authors contributed to the article and approved the submitted version.

## Footnotes

^1^ http://www.R-project.org
