# Supplementary material for: Systemic inflammation mediates environmental polycyclic aromatic hydrocarbons to increase chronic obstructive pulmonary disease risk in United States adults: a cross-sectional NHANES study
Source: Front Public Health. 2023 Nov 21;11:1248812. doi: 10.3389/fpubh.2023.1248812 (PMC10703366; doi:10.3389/fpubh.2023.1248812)
Supplement: Supplementary file 1 [file Data_Sheet_1.docx]

**Table S1. Spearman correlation coefficient**

| **Variables** | **logNAP** | ***P-*value** | **logFLU** | ***P-*value** | **logPA** | ***P*-value** | **logPYR** | ***P-*value** | **logSII** | ***P*-value** |
| --- | --- | --- | --- | --- | --- | --- | --- | --- | --- | --- |
| **logNAP** | 1.000 | 0.000 | 0.666 | 0.000 | 0.552 | 0.000 | 0.513 | 0.000 | 0.017 | 0.274 |
| **logFLU** | 0.666 | 0.000 | 1.000 | 0.000 | 0.777 | 0.000 | 0.764 | 0.000 | 0.43 | 0.006 |
| **logPA** | 0.552 | 0.000 | 0.777 | 0.000 | 1.000 | 0.000 | 0.748 | 0.000 | 0.020 | 0.198 |
| **logPYR** | 0.513 | 0.000 | 0.764 | 0.000 | 0.748 | 0.000 | 1.000 | 0.000 | -0.018 | 0.252 |
| **logSII** | 0.017 | 0.274 | 0.43 | 0.006 | 0.020 | 0.198 | -0.018 | 0.252 | 1.000 | 0.000 |
